# High-Protein Bar as a Meal Replacement in Elite Sports Nutrition: A Pilot Study

**DOI:** 10.3390/foods10112628

**Published:** 2021-10-29

**Authors:** Pavle Jovanov, Marijana Sakač, Mihaela Jurdana, Zala Jenko Pražnikar, Saša Kenig, Miroslav Hadnađev, Tadeja Jakus, Ana Petelin, Dubravka Škrobot, Aleksandar Marić

**Affiliations:** 1 Institute of Food Technology in Novi Sad, University of Novi Sad, Bulevar cara Lazara 1, 21000 Novi Sad, Serbia; marijana.sakac@fins.uns.ac.rs (M.S.); miroslav.hadnadjev@fins.uns.ac.rs (M.H.); dubravka.skrobot@fins.uns.ac.rs (D.Š.); aleksandar.maric@fins.uns.ac.rs (A.M.); 2 Faculty of Health Sciences, University of Primorska, Polje 42, UP 6310 Izola, Slovenia; mihaela.jurdana@fvz.upr.si (M.J.); zala.praznikar@fvz.upr.si (Z.J.P.); sasa.kenig@fvz.upr.si (S.K.); tadeja.jakus@fvz.upr.si (T.J.); ana.petelin@fvz.upr.si (A.P.)

**Keywords:** high-protein bar, quality, sensory properties, dietary intervention study, health beneficial effects

## Abstract

This study was focused on the creation of high-protein bars formulated using whey protein isolate (24%) and soy protein isolate (6%) as the sources of proteins; oat flakes and inulin, both abundant in dietary fibres, and creatine monohydrate and other minor ingredients (vitamin and mineral mixture, potassium sorbate) to achieve the requirements for a meal replacement formula for physically active people. The nutritional profile of the high-protein bar was examined (energy 1215 kJ/288 kcal; protein 34.1 ± 0.20 g, fat 6.01 ± 0.13 g of which was saturated 3.12 ± 0.08 g, fibre 3.10 ± 0.17 g carbohydrate 23.0 ± 0.16 g of which sugars 1.50 ± 0.19 g and starch 21.5 ± 0.11 g in 100 g), and sensory properties with instrumental parameters (texture and colour) were determined and compared with bars commercially available on the market. The created high-protein bar was sensorily acceptable in comparison to other commercially available bars. The dietary intervention study was conducted on elite athletes (professional handball players) to evaluate effects of created versus control bar consumption on their metabolic parameters. The baseline characteristics (mean age, body mass index (BMI), fat mass, muscle mass, lean mass and fat percentage) of the athletes (8) were determined at the start of the study. The cross-over intervention study was organized in two successive phases (5 days each) with a seven-day long washout period between phases. Bars were consumed after the afternoon training unit. Blood samples were collected at the start and the end of the intervention study to analyse the metabolic profiles of the athletes. Serum levels of high-density cholesterol (HDL), low-density cholesterol (LDL) and total cholesterol (HOL), glucose, triacylglycerides (TAG), total and direct bilirubin, creatine kinase (CK), aspartate aminotransferase (AST) and lactate dehydrogenase (LDH) were measured. The results showed that bar consumption significantly decreased serum aspartate transaminase (AST) and lactate dehydrogenase (LDH) and increased total and direct bilirubin levels, suggesting lower exercise-induced muscle damage and increased antioxidative response, respectively. Therefore, it can be concluded that the consumption of the created high-protein bar was able to improve physiological adaptation after training.

## 1. Introduction

The modern lifestyle implies a long working day with changes in eating habits, meaning that traditional meals and their number per day are significantly reduced [1]. Therefore, demands for meal quality, concerning its nutritional and functional profile continually exist. Also, today’s lifestyle is increasingly accompanied by physical activities/sports for supporting health. These activities have to be followed by body protection from mechanical stress and the potential damaging effects of free radicals [2], whose production is reinforced during physical activities/exercise and can lead to oxidative damage of macromolecules [3]. Therefore, the market supply of products such as high-protein bars, initially developed for increasing muscle mass in athletes is increasing [4]. Such products suitably replace consumed energy and nutrients immediately after physical engagement while saving meal preparation time [5].

Although there is a wide range of high-protein bars on the market, they usually contain protein components (20–50 g of high-quality protein per 100 g of the product), sugars and other low weight polyhydroxy compounds (glycerol), alcohols (sorbitol), lipids (palm oil) and other minor ingredients (vitamins, minerals) with a minimal amount of water (water activity—a_w_ ≤ 0.65) [6]. Regardless of the formulations, their creation is still a challenge, primarily because of the interactions among ingredients during mixing, resulting in a product with a fast-changing sensory profile, i.e., a product with a limited shelf life [7,8]. This limitation is a consequence of the product hardening or the development of a tough texture that consumers find unpalatable [9,10]. Texture hardening can be induced by various physical or chemical changes during storage, such as Maillard reactions, sugar crystallization, and molecular migrations. Due to problems related to bar hardening, researchers’ and producers’ focus has to be directed towards the product formula modification [11].

Bovine whey proteins are proven to manifest antioxidant potential in vitro [12], and, therefore, they are expected to provide health benefits to the consumers by combating oxidative stress. Some human intervention studies with whey product supplementation reported increased antioxidant biomarkers in plasma, such as glutathione [13]. Consequently, whey proteins are preferred in high-protein bar formulations to boost body antioxidant defence.

The popularity of creatine supplementation has increased greatly in recent years among athletes due to its performance-enhancing effects [14]. Creatine supplementation has been shown to increase muscle power output and reduce fatigue, ensuring higher intensity and longer duration of the workout. Additionally, its supplementation may positively enhance body composition (gains in muscle mass and possibly fat mass loss) [15].

Utilisation of cereal by-products, e.g., cereal brans, in food formulations can increase the content of total dietary fibre and bioactive compounds such as phenolic acids and flavonoids, oligosaccharides, proteins, folates, sterols, vitamins and minerals [16]. Literature data have shown that oat bran is rich in fibres (18.1–25.2 g/100 g total dietary fibres) [17]. Also, among dietary fibre sources widely used for food enrichment, inulin represents an essential ingredient with a potential beneficial effect on human health, mainly related to its ability to counteract constipation and promote microflora growth in the digestive tract [18].

Due to the reported beneficial effects of the mentioned ingredients, the aims of this work were to (1) produce a high-protein bar using whey protein isolate and soy protein isolate as the sources of proteins, oat bran and inulin representing dietary fibres, creatine monohydrate due to its ability to enhance the energetic system of the body and other minor ingredients (vitamin and mineral mixture, potassium sorbate), which satisfied requirements for a meal replacement formula created for physically active people, (2) examine the nutritional and sensory profile of the produced bar, and (3) evaluate the effects of high-protein bar consumption on metabolic health parameters in elite athletes (professional handball players) throughout a dietary intervention (pilot) study.

## 2. Materials and Methods

### 2.1. Materials

Whey protein isolate, creatine monohydrate and maltodextrin were obtained from Battery Nutrition (Battery Nutrition Limited, London, UK), and soy protein isolate was produced by Sojaprotein (Victoria Group, Bečej, Serbia). Glycerol was obtained from Gram d.o.o (Novi Beograd, Serbia). Oat bran, expanded rice and cocoa butter were purchased from Biouna (Novi Sad, Serbia). Inulin FibrulineTM Instant was purchased from Cosucra Groupe Warcoing S.A (Warcoing, Belgium), the vitamin and mineral mixture was purchased from Maxlab (Novi Sad, Serbia) and potassium sorbate was purchased from Alfa Aesar (Kandel, Germany).

Commercial protein bars were purchased for comparing their characteristics with those of the created high-protein bar. They were selected based on popularity and market availability (Sample 1: energy 1599 kJ/382 kcal, carbohydrate 44 g of which sugars 15 g, protein 25 g; Sample 2: energy 1153 kJ/275 kcal, carbohydrate 38 g of which sugars 16 g, protein 5.2 g; Sample 3: energy 1815 kJ/432.2 kcal, carbohydrate 30 g of which sugars 9.8 g, protein 32 g; Sample 4: energy 1828 kJ/438 kcal, carbohydrate 37.9 g of which sugars 37.6 g, protein 14.2 g).

### 2.2. Preparation of High-Protein Bar

The high-protein bar was produced using the ingredients weighed as follows: whey protein isolate 24 g, glycerol 24 g, maltodextrin 15 g, oat bran 12 g, soy protein isolate 6 g, cocoa butter 6 g, creatine monohydrate 5 g, expanded rice 5 g, inulin 1.33 g, vitamin and mineral mixture (potassium chloride, calcium carbonate, L-ascorbic acid, DL-α-tocopheryl acetate, thiamine mononitrate, riboflavin, nicotinamide, retinyl palmitate, dexpanthenol, pyridoxine hydrochloride, D-biotin, pteroylmonoglutamic acid, sodium phosphate, cyanocobalamin, copper(II) citrate, zinc acetate, iron(II) sulphate, potassium iodide, magnesium oxide, manganese sulphate, sodium selenite, sodium chloride) 1.57 g, and potassium sorbate 0.1 g.

The production process consisted of 3 phases. Phase 1 was mixing cocoa butter and glycerol in a mixing bowl (Bench planetary mixer, Conti, Bussolengo Verona, Italy) to obtain a homogenous mixture. In phase 2, minor (less represented) powdered ingredients were mixed to form a premix (5 min), and in the next step, the premix was mixed with the major ingredients for 10 min using the Bench planetary mixer (Conti, Bussolengo Verona, Italy). In phase 3 the cocoa butter-glycerol mixture was added to the obtained powdered mixture and mixed (75 rpm) for 30 min.

**Figure 1:**
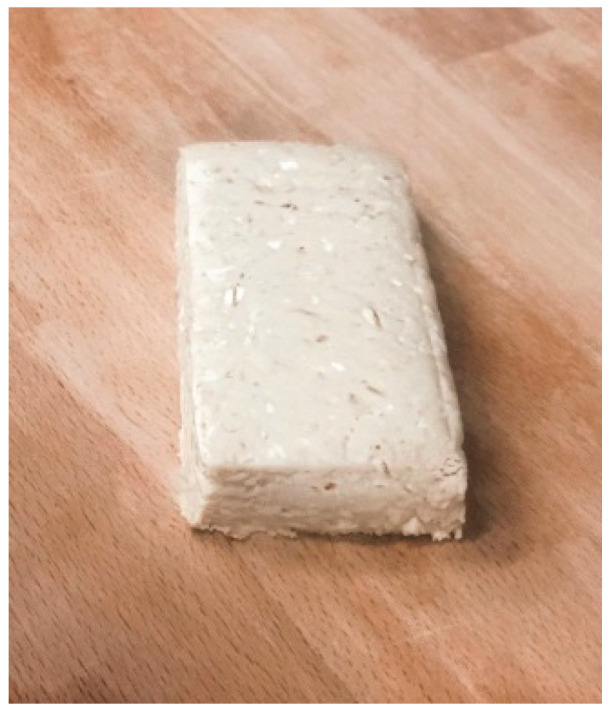
Produced high-protein bar.

After mixing, the mass was rested for 5 min and transferred into an automatic hydraulic divider (Mac.Pan, Thiene, Italy) to form the bars (30 mm × 120 mm inner dimensions). The obtained bars (Figure 1) were packed in 40 μm polypropylene/polypropylene (OPP/OPP) bags and stored at ambient temperature (23 ± 1 °C) before the dietary intervention study.

### 2.3. Proximate Composition and Mineral Content

The proximate composition of high-protein bars including protein (Method No. 950.36), fat (Method No. 935.38), total sugar (Method No. 975.14), total dietary fibre (Method No. 958.29), and moisture contents (Method No. 926.5) was determined by AOAC standard methods of analysis [19]. Starch content was determined by hydrochloric acid dissolution according to the ICC Standard No. 123/1 [20]. Content of NaCl was determined according to SRPS E.Z8.012 [21]. Minerals were determined by atomic absorption spectrophotometry (Method No. 984.27) on a Varian Spectra AA 10 (Varian Techtron Pty Ltd., Mulgvare, Victoria, Australia).

### 2.4. Textural Analysis

The produced high-protein bar together with four commercially available samples were subjected to textural analysis using a TA-XT Plus Texture Analyser (Stable Micro System, Godalming, UK) equipped with a 30-kg load cell and a Craft Knife (A/CKB) suitable for measurement of cutting force, which indicates the hardness of the sample. Bar samples were placed directly on the platform and cut in compression mode using the Craft Knife blade (pre-test speed of 2.5 mm/s, test speed 2 mm/s, and post-test speed 10 mm/s). Maximum force at cutting was registered and represented an indicator of bar hardness.

### 2.5. Colour Measurements

The colour was measured on a top bar cross-section surface using a Minolta Chromameter (Model CR-400, Konica Minolta Co., Osaka, Japan) to obtain CIE L*a*b* coordinates, where L* refers to the lightness (L* = 0 for black, L* = 100 for white), a* refers to the green-red (a* < 0 for green, a* > 0 for red), and b* refers to the blue-yellow (b* < 0 for blue, b* > for yellow). Colour saturation (C*) and colour hue (h*) were measured and compared between the samples. Colour measurements were taken from each sample at five points (1 central and 4 corner points) at three cross-sections.

### 2.6. Sensory Evaluation

Sensory descriptive analysis of the bar samples was conducted by ten experienced and trained sensory panellists (5 females and 5 males, at the age of 25 to 40). All panellists were selected and trained following ISO 8586:2012, respecting all protocols to avoid harm and risks to the participants. The panellists received written information about the study and signed informed consent to participate. Panellists were provided with a list of descriptors, and during training sessions, descriptors’ appropriateness and their definitions were discussed. The final list consisted of eight descriptors which were focused on sweet and bitter taste intensity, odour and flavour intensity and textural properties (cohesiveness, hardness, chewiness and adhesiveness) of the bar samples. The intensities of sensory properties were evaluated on a 100 mm line scale with word anchors at both ends. During two separate sessions, sensory evaluation was performed according to a balanced complete block design (XLSTAT software (Addinsoft, New York, NY, USA)) to avoid order effect. Every panellist was provided with one sample per time in closed odourless plastic containers at ambient temperature (23 ± 1 °C) labelled with three randomly chosen digit numbers and drinking water for palate cleansing. Testing took place in a sensory laboratory equipped with all necessary facilities.

### 2.7. Study Participants

A homogeneous sample consisting of professional handball players was purposively selected for a dietary intervention study. Specifically, eight healthy Caucasian male elite athletes of a male handball team from the First Slovenian Handball League were included in the study through personal contact. The study was conducted during the season with 12 team training units per week (each lasting 1.5 h in the morning and afternoon). In addition to the team training sessions, the players completed 4–5 games per month. Exclusion criteria were infections in the last month and unstable weight in the last three months. Anthropometric and biochemical measurements were performed in the fasting state at 7 a.m. at the University of Primorska, Faculty of Health Sciences, Slovenia. Players provided written informed consent for participation in a scientific assessment. Slovenia’s National Ethics Committee approved all procedures performed in this study (code 0120-557/2017/4). The study was also registered at clinicaltrials.gov (NCT04007731).

### 2.8. Dietary Intervention (Pilot) Study

The randomized, cross-over intervention pilot study was conducted between March 2019 and June 2019. The study was organised into two successive phases to evaluate two protein bars’ metabolic effects—created high-protein bar and a commercially available protein bar (the control bar—Sample 2). The following nutritive parameters characterised the control bar (65 g): energy 1153 kJ/275 kcal, fat 10 g of which saturated 3.7 g, carbohydrate 38 g of which sugars 16 g, fibre 2.3 g, protein 5.2 g and salt 0.4 g. It was selected among commercially available bars based on the similar energy content to the created high-protein bar.

Each study phase lasted five days. After recruitment, the subjects were randomly allocated to one of two intervention groups by a staff member. Four men first consumed the experimental high-protein bar, and in the second intervention period they consumed the control bar, whereas for the other four men it was vice versa (Figure 1). There was a seven-day washout period between the two phases, during which the participants abstained from eating protein bars. The protein bar was always consumed after the afternoon training unit.

**Figure 2:**
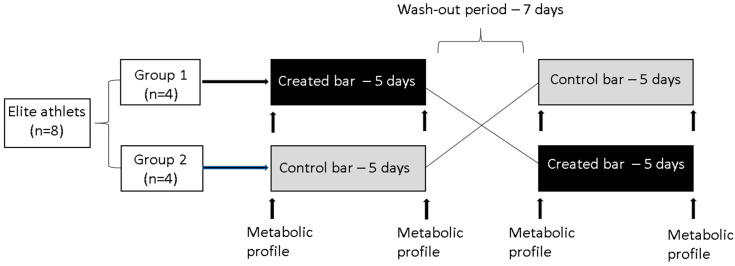
Study protocol for the randomized cross-over study.

Before the study, an expert dietitian assessed the eating and physical activity habits of each participant. The subjects followed some simple rules throughout the study phases to minimize potential confounding variables derived from individual lifestyle. These included intensive physical activity maintenance to 3 h per day and maintenance of usual eating patterns. Food intake and physical activity were monitored throughout the study by 3-day diet records. The expert dietitian carefully explained how to record everything the subjects were eating and drinking and how to record their physical activity. Dietary data were analysed with the Open Platform for Clinical Nutrition (OPEN), accessible through the website http://opkp.si/ (accessed on 21 November 2019). At the start and the end of the intervention, body composition was measured using bioelectrical impedance (Tanita MC-980MA, Maeno-cho, Japan), while the food and exercise diaries were discussed with the subjects. At the same points (Figure 2), venous blood samples were collected to analyse metabolic profile.

### 2.9. Metabolic Assessment

Venous blood samples were collected in vacuum test tubes in the morning between 7. a.m. and 9. a.m. in a fasting state, and serum samples were prepared and frozen until further analysis. Serum levels of high-density cholesterol (HDL), low-density cholesterol (LDL) and total cholesterol (HOL), glucose, triacylglycerides (TAG), total and direct bilirubin, creatine kinase (CK), aspartate aminotransferase (AST) and lactate dehydrogenase (LDH) were measured on a Cobas c111 analyzer (Roche Ltd., Basel, Switzerland) using corresponding Cobass reagents.

### 2.10. Statistical Analysis

The data were processed statistically using the software package XLSTAT 2018.7. All data were averaged and expressed as means ± standard deviation (SD). Analysis of variance (ANOVA) and Tukey’s HSD test for comparison of sample means were used to analyse variations among the textural properties, colour parameters and sensory profiles of the investigated protein bars. A pairwise *t*-test was used to compare values before and after the intervention within each group (the produced bar, the control bar), whereas the comparison of mean changes between the two groups was analysed using an independent *T*-test. Moreover, the interventions’ effects were analysed by a univariate analysis of covariance (ANCOVA) with change from baseline as a dependent variable, adjusted to the corresponding values at baseline (b-model).

## 3. Results and Discussion

### 3.1. Bar Characterisation

The high-protein bar was produced according to the formulation given in Section 2.2, which was prescribed to achieve the following benefits:

- Meal replacement (if the bar package of 100 g is consumed);
- Low energy diet (reduced fat content), especially suitable for people on a weight loss diet/people interested in body weight control;
- Meal with no added sugar;
- Source of dietary fibre;
- Provide a daily need for creatine; and
- Provide the necessary amounts of various vitamins and minerals that should meet the necessary daily intake per meal.

The production of high-protein bar using functional ingredients (protein-rich ingredients, fibre-containing ingredients, creatine, vitamin-mineral mix) resulted in its nutritional and functional profile presented in Table 1.

### 3.2. Textural Analysis

**Figure 3:**
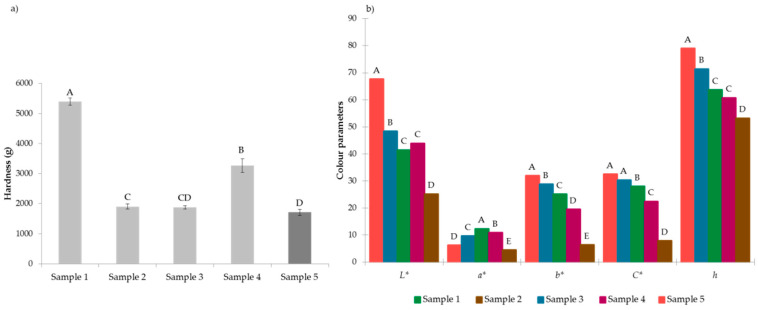
Textural properties (hardness) of protein bars (**a**); colour properties of protein bars (**b**); Sample 1–4: commercially available bars (Sample 1: protein 25.0 g; Sample 2: protein 5.2 g; Sample 3: protein 32.0 g; Sample 4: protein 14.2 g), Sample 5: created high-protein bar: protein 34.0 g. A, B, C, D above the bars indicate significant difference at *p* < 0.05.

Hardness is an important parameter of high-protein nutrition bars. Differences in the hardness of the produced high-protein bar compared to commercially available bars are presented in Figure 3a. The produced high-protein bar was significantly (*p* < 0.05) less hard than the other analysed samples. Due to the physicochemical changes of protein over storage, high-protein bars’ hardness often increases [22]. Therefore, the observed differences in the hardness of the analysed bar samples may have be due to differences in composition and differences in ageing. However, the hardness of the produced high-protein bar was in line with that of other commercially available protein bars. Bearing in mind that texture attributes are essential for consumers’ sensory perception [23], the obtained results suggest that the produced protein bar’s hardness should not be a barrier to consumption.

### 3.3. Colour Measurements

The colour of samples was measured instrumentally, and results are presented in Figure 3b, indicating that the colour of the created high-protein bar was moderately light (L* = 67.6 ± 2.04) with a beige nuance whose hue (h = 79.0 ± 1.13) was more contributed by yellow (b* = 31.9 ± 1.30) and less by red (a* = 6.19 ± 0.65) nuance. The produced high-protein bar possessed significantly (*p* < 0.05) higher lightness, yellowness and colour saturation than the other analysed samples.

Proximate composition and mineral contents were determined as described in Section 2.

### 3.4. Sensory Analysis

**Figure 4:**
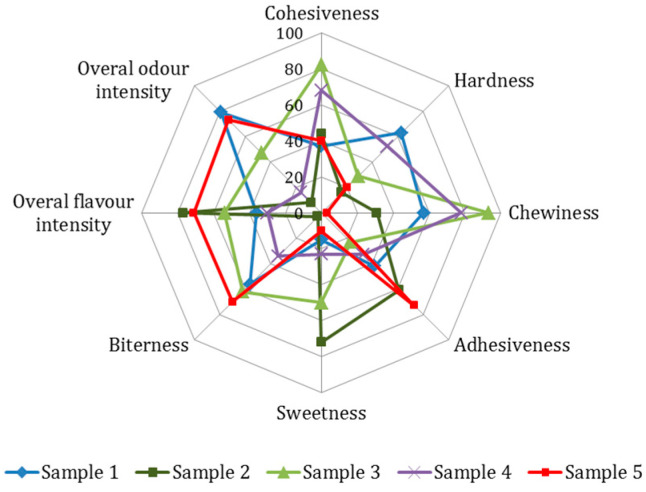
Sensory profile of protein bars. Sample 1–4: commercially available bars (Sample 1: protein 25.0 g; Sample 2: protein 5.2 g; Sample 3: protein 32.0 g; Sample 4: protein 14.2 g), Sample 5: created high-protein bar: protein 34.0 g.

Sensory analysis was conducted to evaluate the created high-protein bar in comparison with four commercially available protein bars. Sensory evaluation of investigated protein bars indicated that the created high-protein bar was moderately cohesive, significantly (*p* < 0.05) less chewy and less hard, as compared to most of the analysed commercial samples (Figure 4). The produced high-protein bar stood out from other evaluated bars in terms of pronounced overall odour and flavour intensity, adhesiveness, and more intense bitter taste. The bitterness of high protein foods may be attributed to the presence of peptides and amino acids [24,25] and, in this particular case, due to lipids present in oats [23]. On the other hand, the more noticeable bitter taste of the created high-protein bar may also be attributed to the fact that this sample was perceived as being significantly (*p* < 0.05) less sweet than the commercial samples, in which bitter taste may be suppressed with the addition of sweet-tasting compounds [26]. According to Pinto et al. [27], the acceptability of the protein bar was decreased due to bitter aftertaste. Bearing in mind that negative taste perception can be barrier to the acceptance of products, further analysis should focus on consumer acceptance, followed by correction of protein bar composition to achieve consumer demands.

### 3.5. Study Population

The baseline characteristics of the eight healthy professional handball players allocated at the start of the study are presented in Table 2. The mean age, body mass index (BMI), fat mass, muscle mass, lean mass and fat percentage were 22.7 ± 2.1 years, 25.3 ± 1.4 kg/m^2^, 14.6 ± 3.7 kg, 73.8 ± 7.1 kg, 77.5 ± 7.5 kg, and 14.6 ± 3.6%, respectively, and the mean visceral fat rating was 2.8 ± 1.5. Food intake and physical activity were monitored by the diaries throughout the study. The average daily energy intake was 2710 ± 611 kcal, while the daily intake of protein, carbohydrate and total fat was 129 ± 58 g, 298 ± 99 g and 105 ± 17 g, respectively. Overall, there were no statistically significant differences in daily energy intake between the two groups at baseline and after the intervention period of the two study phases (data not shown). There were no differences in physical activity between the players. They were physically active for 3 h per day.

### 3.6. Effects of Experimental and Control Bar Consumption on Metabolic Profile

**Figure 5:**
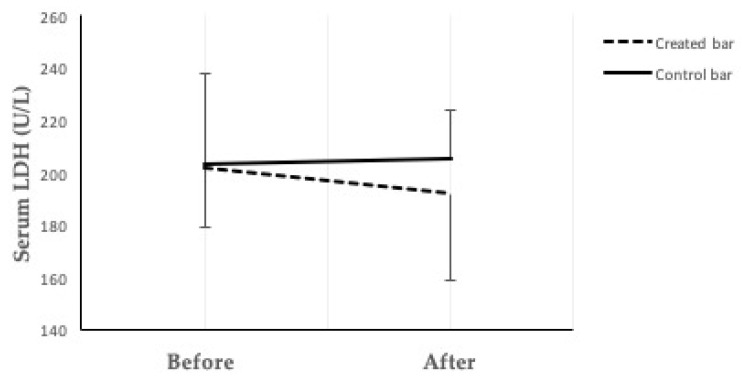
Serum LDH concentration before and after the created bar (dashed black lines) and the control bar (solid black lines) intake. Values are presented as means ± SD.

The effects of the consumption of protein bars on serum glucose levels, lipid profile, inflammatory marker C-reactive protein (CRP), anti-inflammatory and antioxidant bilirubin, liver enzyme aspartate transaminase (AST), and the markers of muscle damage creatine kinase (CK) and lactate dehydrogenase (LDH) were analysed (Table 3). A pairwise t-test revealed no significant effect of either of the two bars (created and control). However, when the initial value of the measured parameter was considered as a covariate, changes in serum levels of both total and direct bilirubin were found to be significantly increased (*p* = 0.017; *p* = 0.049, respectively) when participants consumed the created high-protein bar. Also, the decrease in serum level of liver enzyme AST was slight but significant. The level of LDH significantly decreased from 202 ± 23 to 192 ± 33 U/L (*p* = 0.044) (Figure 5). In the case of the control bar consumption, neither the pairwise *t*-test nor the use of a statistical model revealed any statistically significant change. There was also no statistically significant difference between the two groups. Based on the results presented in Table 3, the created bar could increase total and direct bilirubin levels and mitigate AST and LDH levels compared to the same physiological parameters obtained after the consumption of the control bar.

Improvement in muscle mass and exercise performance, fat loss, and improvement in recovery biomarkers are the basic health benefits result from protein supplement consumption [28]. During prolonged or high-intensity exercise, cell membrane permeability is increased, and various enzymes, including AST and LDH, may be released, indicating muscle damage [29]. The present 5-day long pilot study demonstrated the short-term effects of the created high-protein bar consumption on AST and LDH serum marker reduction, i.e., its potential to protect athletes’ exercise-induced muscle damage.

Many nutritional supplements are effective in various experimental models in terms of their protective effects on these biomarkers [30,31,32]. In a study of acute effects, whey protein hydrolysate showed more remarkable improvement of muscle damage markers compared to carbohydrate drink [33]. Unlike the mentioned result, most studies evaluating the acute effects of a single protein meal intake after exercise, compared to a carbohydrate meal, found no difference in parameters related to muscle regeneration [34,35]. This suggests that beneficial effect may not be detected immediately after a one-time consumption, pointing to a slower mechanism.

Circulating serum bilirubin (total and direct bilirubin) is a by-product of normal blood metabolism that contributes to the assessment of liver function and the extent of haemolysis and is often used as a biomarker for cholestasis [36]. In the present study, bilirubin was found to increase within the accepted reference limit, indicating normal liver function and no significant effect on exercise-induced haemolysis. Moreover, several recent animal and human studies confirm that bilirubin is a potential anti-inflammatory and antioxidant agent that protects us from free radical damage and is inversely correlated with many types of disease [37,38]. It also inhibits oxidative changes in LDL and other lipids, participates in the neutralisation of free radicals and prevents oxidative stress, which can be induced by oxygen consumption during muscle activity [39]. Therefore, the moderate increase in serum bilirubin levels detected in this study (Table 3) may be a compensatory mechanism through which the overall oxidative stress was lowered and thus beneficial for athletes. This means that the created high-protein bar can ameliorate muscle damage and promote muscle recovery.

Inulin was added to the created bar formulation due to its known positive effects on gut microbiota [40], and intestinal wall integrity [41], which could lead to reduced inflammation. No differences in the inflammatory marker CRP were detected in our study, most probably because the positive effects would only be visible after longer-term consumption. An extended intervention study (6–8 weeks long) on a larger sample could be recommended based on the observed positive effects in this pilot study.

Based on the metabolic profiles of professional handball players presented in Table 3, it can be concluded that the created high-protein bar, when consumed immediately after training, provided physiological protection for handball players.

## 4. Conclusions

The high-protein bar was created using whey and soy protein isolate as the sources of proteins, oat flakes and inulin, both abundant in dietary fibres, creatine monohydrate and other minor ingredients (vitamin and mineral mixture, potassium sorbate) to meet the criteria for a meal replacement formula for physically active people. The created bar possessed a similar sensory profile (texture, colour, and sensory properties) compared to commercially available high-protein bars. The metabolic profiles of professional handball players obtained in the dietary intervention study in which the effects of the created high-protein bar consumption on their health status were monitored suggested that post-exercise consumption reduced oxidative stress and positively influenced recovery from exercise-induced muscle damage.

## Figures and Tables

**Table 1:** The nutritional profile of the high-protein bar.

Parameter	100 g
Energy	1215 kJ/288 kcal
Moisture (g)	6.08 ± 0.05
Fat (g)of which saturated	6.01 ± 0.133.12 ± 0.08
Carbohydrate (g)of which sugars	23.0 ± 0.161.50 ± 0.19
Starch (g)	21.5 ± 0.11
Fibre (g)	3.10 ± 0.17
Protein (g)	34.1 ± 0.20
NaCl (g)	0.51 ± 0.07
**Active ingredients**	**100 g**
Creatine-monohydrate (mg)	5000
Vitamin A (µg RE *)	210 (26% **)
Vitamin D (µg)	1.5 (30% **)
Vitamin E (mg-α-TE ***)	3 (25% **)
Vitamin C (mg)	13.5 (17% **)
Thiamin (mg)	0.33 (30% **)
Riboflavin (mg)	0.48 (34% **)
Niacin (NE)	5.4 (34% **)
Vitamin B_6_ (mg)	0.45 (32% **)
Folate (µg)	60 (30% **)
Vitamin B_12_ (µg)	0.42 (17% **)
Biotin (µg)	7.5 (15% **)
Pantothenic acid (mg)	0.9 (15% **)
Calcium (mg)	210 (26% **)
Phosphorus (mg)	165 (24% **)
Potassium (mg)	930 (46% **)
Iron (mg)	2.10 (15% **)
Zinc (mg)	2.85 (28% **)
Copper (mg)	0.33 (33% **)
Iodine (µg)	42.9 (29% **)
Selenium (µg)	18.2 (33% **)
Sodium (mg)	173
Magnesium (mg)	56.35 (15% **)
Manganese (mg)	0.31 (15% **)

* RE—all trans retinol equivalent; ** NRV—nutrient reference values; *** α-TE—alpha-tocopherol equivalents; vitamin contents were calculated from the declaration of the purchased vitamin and mineral mixture. Creatine content was obtained by calculation using the declared content on the creatine monohydrate product label.

**Table 2:** Basic characteristics of professional handball players who participated in the dietary intervention (pilot) study.

Variable	Mean ± SD
Age (years)	22.7 ± 2.1
BMI (kg/m^2^)	25.3 ± 1.4
Fat mass (kg)	14.6 ± 3.7
Muscle mass (kg)	73.8 ± 7.1
Lean mass (kg)	77.5 ± 7.5
Fat (%)	14.6 ± 3.6
Visceral fat rating	2.8 ± 1.5
Energy intake (kcal/day)	2710 ± 611
Proteins (g/day)	123 ± 58
Carbohydrates (g/day)	298 ± 99
Total fat (g/day)High intensity physical activity (h/day/MET-h/day)	105 ± 17 3/11

Values are means of three determinations ± standard deviation. BMI: body mass index.

**Table 3:** Effects of the created and the control bar consumption on the metabolic profile of professional handball payers ^1^.

Created Bar Intake					Control Bar Intake				
	Before	After	p^a *t*-test^	p^b model^	Before	After	p^a *t*-test^	p^b model^	p^c-index.^
**Biochemical Parameters**									
CK (U/L)	327.6 ± 197.1	352.6 ± 351.5	0.732	0.325	446.5 ± 271.1	348.8 ± 183.0	0.207	0.075	0.242
HOL (mmol/L)	3.70 ± 0.56	3.62 ± 0.68	0.385	0.337	3.72 ± 0.44	3.72 ± 0.57	0.987	0.475	0.705
LDL (mmol/L)	2.33 ± 0.70	2.27 ± 0.70	0.289	0.359	2.18 ± 0.47	2.20 ± 0.70	0.799	0.578	0.500
HDL (mmol/L)	1.28 ± 0.46	1.21 ± 0.31	0.373	0.238	1.44 ± 0.62	1.46 ± 0.47	0.787	0.747	0.452
TAG (mmol/L)	1.12 ± 0.43	1.18 ± 0.28	0.601	0.084	1.10 ± 0.47	1.18 ± 0.53	0.767	0.772	0.925
Glucose (mmol/L)	4.90 ± 0.26	4.97 ± 0.44	0.682	0.515	4.98 ± 0.40	5.26 ± 0.60	0.06	0.212	0.306
CRP (mg/L)	0.30 ± 0.32	0.36 ± 0.33	0.992	0.184	0.51 ± 0.53	0.56 ± 0.62	0.858	0.229	0.305
Bilirubin (µmol/L)	12.7 ± 7.4	13.96 ± 4.30	0.416	0.017	11.0 ± 4.5	11.5 ± 7.7	0.813	0.811	0.784
Bilirubin/d (µmol/L)	2.58 ± 1.22	2.80 ± 0.76	0.410	0.049	2.45 ± 0.89	2.5 ± 1.3	0.904	0.834	0.705
AST (U/L)	30.0 ±10.1	28.2 ± 10.1	0.656	0.016	31.2 ± 9.2	28.0 ± 4.5	0.135	0.068	0.815
LDH (U/L)	202 ± 23	192 ± 33	0.277	0.044	203 ± 35	205 ± 19	0.846	0.438	0.349

CK—creatine kinase; HOL—total cholesterol; LDL—low-density cholesterol; HDL—high-density cholesterol; TAG—triacylglycerides; CRP—C-reactive protein; AST—aspartate aminotransferase; LDH—lactate dehydrogenase. ^1^ Values are presented as means ± SD. p^c^-value denotes comparison of mean changes between the 2 groups using an independent *t*-test. p^a^-value denotes differences within the 2 groups using a paired *t*-test. p^b^-value denotes differences within the 2 groups using univariate analysis of covariance with the change from baseline as a dependent variable, adjusted to the corresponding values at baseline.
